# Free and Conjugated Phenolic Profiles and Antioxidant Activity in Quinoa Seeds and Their Relationship with Genotype and Environment

**DOI:** 10.3390/plants10061046

**Published:** 2021-05-21

**Authors:** Fabiana Antognoni, Giulia Potente, Stefania Biondi, Roberto Mandrioli, Lorenzo Marincich, Karina B. Ruiz

**Affiliations:** 1 Department for Life Quality Studies, Rimini Campus, Alma Mater Studiorum—University of Bologna, Corso d’ Augusto 237, 47921 Rimini, Italy; giulia.potente@unibo.it (G.P.); roberto.mandrioli@unibo.it (R.M.); lorenzo.marincich2@unibo.it (L.M.); 2 Department of Biological, Geological and Environmental Sciences, Alma Mater Studiorum—University of Bologna, Via Irnerio 42, 40126 Bologna, Italy; stefania.biondi@unibo.it; 3 Química y Farmacia, Facultad de Ciencias de la Salud, Universidad Arturo Prat, Av. Arturo Prat Ch. 2120, Iquique 1100000, Chile; karuiz@unap.cl

**Keywords:** antioxidant activity, *Chenopodium quinoa*, conjugated phenolics, flavonoids, phenolic acids, nutraceutical properties

## Abstract

The nutraceutical interest in quinoa (*Chenopodium quinoa* Willd.) seeds is associated with the presence of macronutrients, micronutrients, minerals, vitamins, and polyphenols. In particular, polyphenols contribute to the health-promoting effects of this food crop, and their levels are influenced by environmental conditions. Production of quinoa is recently being explored in temperate climate areas, including Italy. The aim of this research was to assess the profile of bioactive compounds in seeds of two quinoa varieties, Regalona-Baer and Titicaca, grown in northern Italy, compared to that of seeds of those varieties grown in Chile and Denmark, respectively. High-performance liquid chromatography-diode array detector (HPLC-DAD) analysis of phenolic acid and flavonoid profiles, both in their free and soluble conjugated forms, showed that the main differences between Regalona grown in Chile and Italy were for the free vanillic acid and daidzein contents, while the two Titicaca samples mainly differed in quercetin derivative levels. The total phenolic index was comparable in Titicaca and Regalona, and only a slight decrease in this parameter was found in seeds of the two varieties grown in Italy. The in vitro antioxidant activity of seed extracts, evaluated by means of three different assays, indicated that it correlated with flavonol (quercetin derivative) levels. In conclusion, the results indicate that, although environmental conditions alter the polyphenolic profile and biological activities, it is possible to grow good-quality quinoa in northern Italy.

## 1. Introduction

Quinoa (*Chenopodium quinoa* Willd.) seeds have an excellent nutritional profile [1,2], due to their remarkably high protein content, well-balanced content of essential amino acids comparable, in terms of quality, to that of casein [3], and the presence of dietary fiber, vitamins, and unsaturated fatty acids, e.g., linoleic and α-linolenic acids [2,3,4,5,6]. Quinoa seeds are considered an excellent example of a “functional food”, able to exert health-promoting effects also due to the presence of secondary metabolites [6]. These include phenolic acids and flavonoids [7], as well as terpenoids, steroids, and large amounts of α- and γ-tocopherol, which are known antioxidants [8]. For some of these specialized metabolites, a clear role in preventing various human diseases, such as neurological and other chronic disorders, has been recognized, as a result of their effects on cell signalling and metabolism [6,9,10].

Quinoa cultivation remained, for a long time, exclusive to Andean populations, especially from Bolivia and Peru [11], and, to a lesser extent, from Chile and Ecuador. The wide genetic diversity of this species, resulting from its fragmented and localized cultivation over the centuries in the Andean region, together with its high tolerance to extreme environmental conditions and abiotic stresses [12,13], has led to the differentiation of five major ecotypes, based on their ability to adapt to specific agroecological conditions [11]. Nevertheless, in recent decades, with the boom in demand for “superfoods”, the interest in this ancient Andean halophytic seed-producing crop has become global, and its cultivation has been growing accordingly. In less than twenty years, the global demand for quinoa grew to such an extent that it led to the triplication of the Andean areas dedicated to its cultivation [14]. Moreover, the adaptability and stress tolerance of this plant is being exploited to establish cultivations outside the Andean territories [11,15]. As a consequence, the number of countries growing quinoa has rapidly risen starting from the 1980s, and, nowadays, more than 95 countries are cultivating or testing quinoa as a crop [16]. In the early 1990s, quinoa began to be cultivated in Europe, and the crop has been successfully tested in several Mediterranean countries, such as Greece, Morocco, Spain, and Italy [17,18,19,20].

The main concern about the introduction of this crop was related to its high sensitivity to photoperiods during the reproductive phase [21]. The best results were achieved with the sea level/coastal ecotype from central and southern Chile, since it is the best adapted to temperate environments. Using these Chilean quinoa lines, a new variety, called Titicaca, was bred in Denmark [22]. Titicaca is one of four European quinoa cultivars (with Puno, Jessie, and Zeno) originating from different gene pools and already tested in various countries, including Germany [23] and Italy, where several field trials have been carried out at different latitudes to test the adaptability of the crop to varying environments [21,23,24]. Pulvento et al. [25] reported good plant performance and tolerance to high temperatures and water deficit, typical conditions of southern Italy, for cultivars Regalona-Baer and Titicaca.

Several studies point to the importance of environmental and/or agronomical factors in affecting the nutritional properties of quinoa. For example, Reguera et al. [26] showed that amino acid profiles, total protein content, mineral composition, and phytate content varied in seeds of three cultivars (Salcedo-INIA, Titicaca, Regalona) grown in three different countries (Spain, Peru, and Chile). However, the impact of agroecological conditions and crop management techniques on quinoa seed quality in terms of functional bioactive compounds (e.g., phenolics) has been little explored. Miranda et al. [27] reported that seeds of several quinoa genotypes grown in different geographical locations in Chile contained variable amounts of phenolics and flavonoids. Considering that these secondary metabolites are plastically produced in plant tissues as an acclimation response to environmental cues, their concentrations in plant tissues are the combined result of several factors, including genetic components, environmental conditions, and the complex interplay between the two [28,29,30]. In the present work, we checked this hypothesis by analysing the phenolic profiles of quinoa seeds of the same variety from different agroecological environments and verified if the antioxidant activity (AA) of seed extracts likewise changed. Such analyses are useful to identify the environmental conditions that modulate the health-promoting characteristics of quinoa seeds. To this purpose, we evaluated the composition in bioactive free and soluble-conjugated phenolic acids and flavonoids and the AA of seeds of two quinoa varieties, the Chilean Regalona-Baer and the Danish-bred Titicaca, grown in northern Italy and compared them with those of seeds of the same varieties grown in Chile and Denmark. The present results could help to establish the best conditions (genotype/geographical cultivation zone) leading to seed enrichment in functional compounds and to reinforce the notion that good-quality quinoa can be grown in Italy.

## 2. Results and Discussion

### 2.1. Free and Soluble-Conjugated Total Phenolic Content (TPC) and Total Flavonoid Content (TFC) in Quinoa Seed Extracts

Phenolic acids and flavonoids in seed extracts were quantified by analysing the free and soluble-conjugated forms (and not the insoluble ones) because they represent the fractions of dietary phenolic compounds that are more rapidly absorbed by the stomach and small intestine and for which important health benefits have been reported [31].

**Figure 1:**
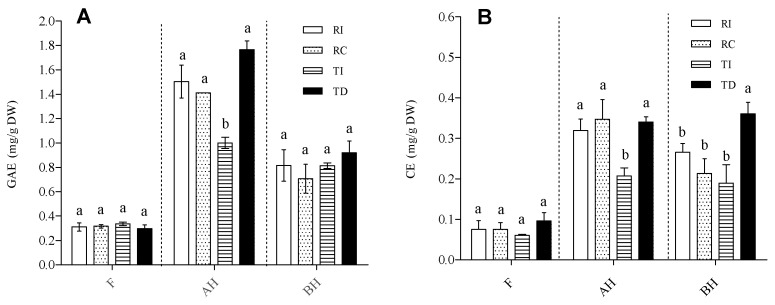
Total phenolic content (**A**) and total flavonoid content (**B**) in free (F), acid-hydrolysed (AH), and base-hydrolysed (BH) fractions of quinoa seed extracts. (**A**) TPC is expressed as mg gallic acid equivalents (GAE)/g DW seed powder. (**B**) TFC is expressed as catechin equivalents (CE)/g DW seed powder. RI: Regalona grown in Italy; RC: Regalona grown in Chile; TI: Titicaca grown in Italy; TD: Titicaca grown in Denmark. Data are the means ± standard error of two independent determinations with three biological replicates. Different letters within the same parameter indicate statistically significant differences at *p ≤* 0.05.

Total phenolic content (TPC) and total flavonoid content (TFC) were evaluated in extracts of the four seed samples enriched in the solvent-extractable (free; F) and in both the acid-hydrolysed (AH) and base-hydrolysed (BH) soluble-conjugated forms (Figure 1).

As concerns TPC (Figure 1A), AH was the richest fraction, with levels reaching 1.7–1.8 mg GAE/g DW of seed powder, followed by the BH fraction (0.6–0.8 mg GAE/g DW), while much lower levels were observed in the free form (0.2–0.3 mg/g DW). In both the F and BH fractions, no significant differences in TPC were observed between RI, RC, TI, and TD. By contrast, the Danish-grown Titicaca seeds (TD) had about twice the concentration of AH soluble-conjugated phenolics as compared with the same cultivar grown in Italy. As regards Regalona, the TPC in this fraction (AH) was very similar in both Chilean- and Italian-grown seeds, and not significantly different from that of TD (Figure 1A). This suggests that the conjugated form of soluble phenolics is mainly linked through ether bonds, such as the glycosidic one, which is acid hydrolysable. The distribution of TPC into the three fractions in the quinoa seeds analysed here is in line with that reported by Tang et al. [32] in a white seed quinoa, but not with that by Gómez-Caravaca et al. [33] for genotypes Kancholla and Witulla. These authors reported that most phenolic compounds present in their seed extracts were in the free form, but it should be underlined that these authors used a different analytical method, i.e., HPLC-DAD-ESI-TOF-MS, which could explain the discrepancies. Although Repo-Carrasco-Valencia et al. [7] observed a huge variation in the proportion of soluble (free and conjugated) phenolics (ranging from 7 to 61%), among the ten genotypes taken into consideration, we did not observe large differences in the total amounts of soluble phenolics between the two T and R varieties. It should be pointed out that the TPC in the AH fraction might be overestimated, since several authors [32,34] indicated that, under acid hydrolysis, the degradation of free sugars can give rise to furan derivatives, which react with the Folin–Ciocalteu’s reagent. Thus, the distribution of phenolic compounds between the free and AH conjugated forms, resulting from the TPC determination, only provides a rough estimate, which must be confirmed through more specific analytical techniques.

As concerns TFC, both the relative distribution in the three forms and the pattern within each fraction were similar to those of TPC; the conjugated forms were more abundant than the free ones, even though, in this case, the levels of AH and BH soluble flavonoids were comparable (Figure 1B). In plants, it is known that flavonoids are mostly conjugated to various types of molecules. The abundance of flavonoids in both the AH and BH fractions indicates that these were linked via both ether and ester bonds; in the former category, glycosides represent the most widespread form, while the latter suggest their linkage to organic acids and proteins, as demonstrated by Koistinen et al. [35]. As concerns the comparison among samples, the pattern of TFC in the free and AH fractions was very similar to that of TPC, with AH forms again higher in TD than in TI. Danish-grown Titicaca showed the highest TFC in the BH fraction, while no differences were found between the other three samples (Figure 1B). Based on these results, both genotype and environmental conditions seem to exert their effect mainly on the conjugated form of phenolics, leaving the free form almost unchanged. Moreover, they indicate that TI had the lowest level of phenolics conjugated through ether linkages. 

### 2.2. Chromatographic Analysis of Free and Soluble-Conjugated Phenolic Compounds in Quinoa Seed Extracts

**Figure 2:**
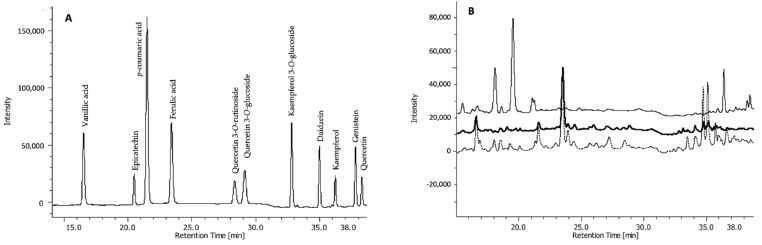
Chromatogram of HPLC-DAD separation of (**A**) standard phenolic acids and flavonoids, and (**B**) representative chromatographic profiles of free (upper line), base-hydrolysed (middle line), and acid-hydrolysed (bottom line) fractions of one seed extract (TI).

The three fractions were subjected to high-performance liquid chromatography-diode array detector (HPLC-DAD) analysis to identify and quantify single phenolic compounds. These include phenolic acids (both hydroxycinnamic and dihydroxybenzoic acids), flavonols, flavan-3-ols, and isoflavones (Figure 2).

**Figure 3:**
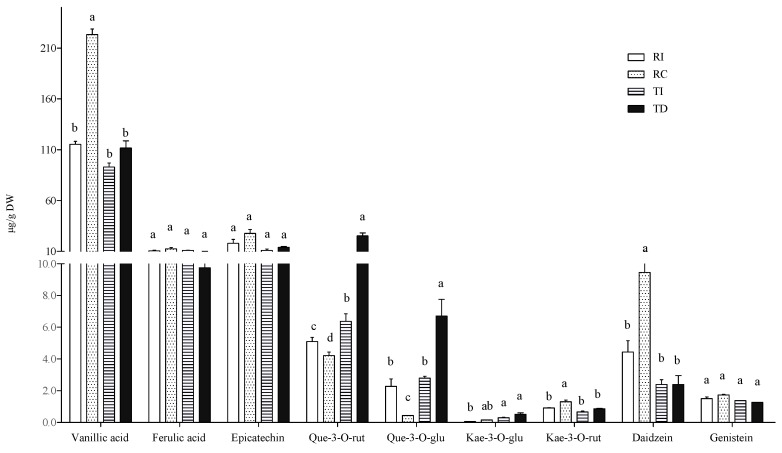
Free phenolic acid and flavonoid concentrations in quinoa seed extracts. RI: Regalona grown in Italy; RC: Regalona grown in Chile; TI: Titicaca grown in Italy; TD: Titicaca grown in Denmark. Data are the means ± standard error of two independent determinations with three biological replicates. Different letters within the same parameter indicate statistically significant differences at *p ≤* 0.05.

In the free fraction, vanillic and ferulic acids were the major hydroxycinnamic acids detected in all samples, the former being about 10-fold more concentrated than the latter (Figure 3). Seed extracts of Chilean Regalona had a vanillic acid content about twice that of the same genotype grown in Italy, and of both TD and TI, whose concentrations were very similar. As for ferulic acid levels, no differences were observed between the two cultivars, no matter where they were grown (Figure 3).

The prevalence of vanillic acid as the main representative of the phenolic acid class in the free form is in line with the results of Tang et al. [32] in a white seed quinoa, even though significant variations in the relative amounts of single phenolic acids in different quinoa genotypes and varieties were also reported by Repo-Carrasco-Valencia et al. [7]. Nevertheless, a comparison with the results from these authors is not easy, since they reported the soluble phenolic acid content without distinguishing between the free and the soluble-conjugated fraction. 

As concerns flavonoids, epicatechin, quercetin, and kaempferol derivatives (quercetin-3-*O*-rutinoside, quercetin-3-*O*-glucoside, kaempferol-3-*O*-glucoside, and kaempferol-3-*O*-rutinoside) and the isoflavones daidzein and genistein were detected in the free fraction (Figure 3). Significant differences among samples were observed only in some cases. In particular, TD seeds showed higher levels of quercetin-3-*O*-glucoside and quercetin-3-*O*-rutinoside compared to seeds of the same cultivar grown in Italy (two- and four-fold, respectively), and compared to both Regalona extracts. In Regalona seeds, a higher level of these two quercetin derivatives was found in Italian samples as compared to Chilean ones. Conversely, the latter showed the highest daidzein levels among all samples, and no differences in this isoflavone were found among other seeds. All seed extracts contained very low amounts of kaempferol derivatives, whose differences were of no biological relevance, nor were any differences observed as concerns genistein and epicatechin (Figure 3). Thus, the agroclimatic conditions in which the Chilean Regalona seeds were produced seem to be particularly favourable for the accumulation of daidzein since, in this sample, the highest daidzein/genistein ratio (=6) was found. This value is very close to that observed by Lutz et al. [36] in some commercial quinoa seeds and suggests that, under certain environmental conditions, a seed with a good phytoestrogen-like activity can be obtained. 

**Figure 4:**
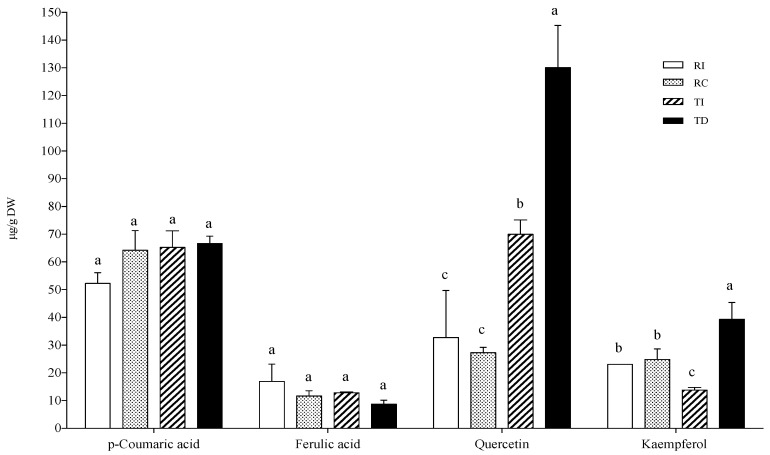
Phenolic acid and flavonoid concentrations in the acid-hydrolysable soluble-conjugated fraction of quinoa seed extracts. RI: Regalona grown in Italy; RC: Regalona grown in Chile; TI: Titicaca grown in Italy; TD: Titicaca grown in Denmark. Data are the means ± standard error of two independent determinations with three biological replicates. Different letters within the same parameter indicate statistically significant differences at *p ≤* 0.05.

In the AH conjugated fraction, *p*-coumaric and ferulic acids did not vary considerably among samples (Figure 4). The ferulic acid concentration was very similar to that found in the free fraction, while *p*-coumaric acid was not detected in the free form. As concerns flavonoids, the aglycones quercetin and kaempferol were found as the main representatives, and TD was confirmed to be the richest in these flavonols (about three-fold higher than in TI), while no differences were detected between the two Regalona samples (Figure 4). 

**Figure 5:**
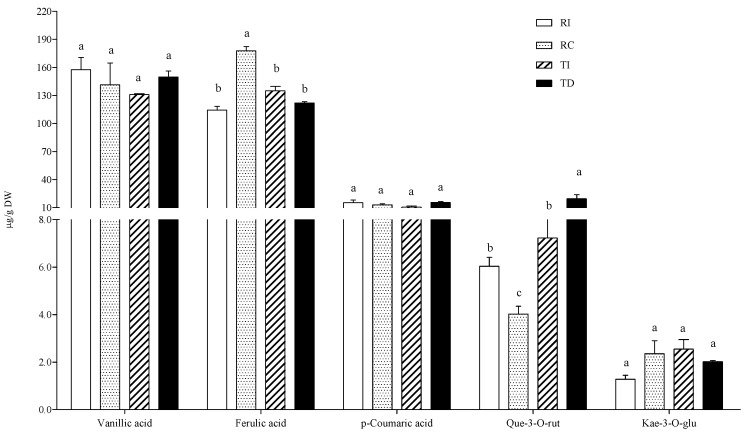
Phenolic acid and flavonoid concentrations in the base-hydrolysed solubleconjugated fraction of quinoa seed extracts. RI: Regalona grown in Italy; RC: Regalona grown in Chile; TI: Titicaca grown in Italy; TD: Titicaca grown in Denmark. Data are the means ± standard error of two independent determinations with three biological replicates. Different letters within the same parameter indicate statistically significant differences at *p* ≤ 0.05.

Phenolic acids and flavonoids were also detected in the BH soluble conjugated fraction. Both *p*-coumaric and ferulic acids were present, and the latter was detected at higher levels than in the AH fraction (Figure 5).

Vanillic acid was present at similar levels to those of the free fraction, while conjugated ferulic acid was over ten-fold more concentrated than in the free form. Thus, the results indicate that a relevant portion of these polyphenols is conjugated through ester bonds with soluble cellular components, such as short peptides and/or low-molecular weight oligosaccharides. It is known that ferulic and *p*-coumaric acids are covalently bound to mixed-linkage (1→3, 1→4)-β-D-glucans and to hemicelluloses, forming the soluble dietary fiber [37]. Being a dicot species, quinoa cell walls contain mainly xyloglucans in their matrix. The extractability of hemicelluloses during the preparation of crude seed extracts using sonication has been demonstrated [38] as well as the binding of ferulic acid to short-chain hemicelluloses [39]. Thus, the crude extracts used in this study probably contain soluble feruloylated and/or coumaroylated xylo-oligosaccharides, which could explain the presence of high levels of aglycone components in our BH fraction. Several health-promoting activities have been reported for these short-chain feruloylated oligosaccharides, including an immunomodulatory effect [40]. As concerns the comparison among samples, the results indicate that concentrations of vanillic and *p*-coumaric acids did not differ significantly between Regalona and Titicaca, no matter where they were grown, while for ferulic acid, a 55% higher concentration was found in RC compared to seeds of the same genotype grown in Italy; no differences were found between it and TD or TI seeds. 

Quercetin-3-*O*-rutinoside (or rutin) and kaempferol-3-*O*-glucoside were identified as the main flavonols in the BH fraction. As regards the former, the pattern was similar to those in the free and AH conjugated fractions, with DT being richer than the Italian-grown counterpart, while RI had a slightly, but significantly, higher rutin content than RC; no differences were observed for kaempferol-3-*O*-glucoside among the four samples (Figure 4).

Some authors have investigated the phenolic composition of different quinoa varieties, and phenolic acids and flavonoids were reported to exist both in their aglycone and glycosidic forms [32,41,42]. On average, the concentrations of these compounds found in our work were similar to those found by other authors [41,43], but lower compared to other reports [33]. These discrepancies are not surprising, since several parameters were demonstrated to have a great impact on the extraction efficiency of these metabolites from plant material, such as sampling, solvent-to-solid ratio used for extraction, duration of extraction, and sonication treatment applied, among others [44]. The use of more advanced separative techniques, such as the HPLC-DAD-ESI-TOF-MS utilized by Gómez-Caravaca et al. [33], allowed these authors to identify more compounds, which are not detectable with other techniques, and this can also explain the differences. 

Hirose et al. [45] reported that quinoa and buckwheat contain several quercetin and kaempferol derivatives, which are not present in any of the widely consumed cereals. They also compared the flavonoid pattern and in vitro antioxidant capacity of seeds cultivated in Japan with those of seeds from Bolivia and Peru. Results indicated that the major differences between the two groups of seeds were found for quercetin and kaempferol derivatives, which were significantly higher in Japanese-grown seeds compared to South American ones, and this was accompanied by a significantly higher antioxidant capacity. Seasonal variations in the pattern of flavonol glycosides observed by these authors allowed them to hypothesize that sunlight was the factor that mostly influenced the accumulation of quercetin glycosides [45]. Thus, it is possible that variations in quercetin and its derivatives between Titicaca and Regalona grown at different latitudes may be due to differences in day length and light quality/intensity. Even though the effect of latitude on flavonoid accumulation has yet to be fully clarified, due to the complexity of latitude itself as a parameter to be investigated [29], many studies have concluded that northern climates may have a positive impact on the biosynthesis of flavonoids in plants, although there are variations in the response among species and within individual flavonoid groups. Flavonols, especially moieties of quercetin or kaempferol, have been reported to accumulate in response to increased UV-B radiation [46], which typically characterizes the higher latitudes in the Northern Hemisphere. The prominent role for this subclass of flavonoids in regulating plant–environment interactions has been demonstrated, particularly as concerns the acclimation of plants to different light exposures [47]. In general, flavonoids may play prominent roles as scavengers of reactive oxygen species (ROS) generated by adverse conditions [48,49], and quercetin derivatives, due to their chemical characteristics, are particularly efficient in buffering alterations in ROS homeostasis [28]. Moreover, several reports indicate that they strongly affect phytohormone (auxin and ABA) signalling, due to their ability to affect the activity of a wide range of proteins [50,51]. Thus, the higher accumulation of quercetin derivatives in Titicaca seeds grown in Denmark compared to seeds of the same cultivar grown in Italy and to both Regalona samples might be the result of an acclimation mechanism to long light exposures, typical of the higher latitudes. The bioavailability and therapeutic potentials of quercetin and its derivatives in plant-based foods have been extensively investigated [52], and several studies demonstrated that they play relevant roles both in the prevention and treatment of chronic diseases, including cardiovascular and neurodegenerative ones and some types of cancer [53,54,55]. Thus, the enrichment of quinoa seeds in flavonol derivatives could represent an added value able to reinforce the well-known nutraceutical properties of this plant.

### 2.3. Antioxidant Activity of Quinoa Seed Extracts

**Figure 6:**
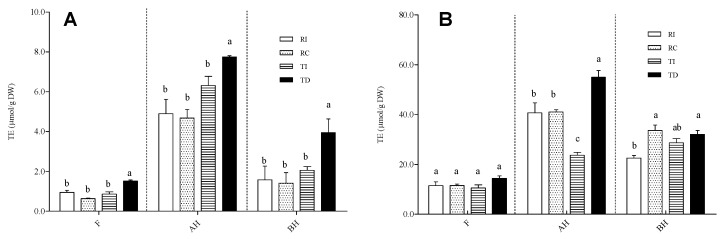
Antioxidant activity, assayed by TEAC (**A**) and ORAC (**B**), in free (F), acid-hydrolysed (AH), and base-hydrolysed (BH) fractions of seed extracts from different quinoa samples. Results are expressed as Trolox equivalents (TE)/g DW seed powder. RI: Regalona grown in Italy; RC: Regalona grown in Chile; TI: Titicaca grown in Italy; TD: Titicaca grown in Denmark. Data are the means ± standard error of two independent determinations with three biological replicates. Different letters within the same parameter indicate statistically significant differences at *p* ≤ 0.05.

The AA of the three fractions of Regalona and Titicaca seed extracts was evaluated using three different in vitro assays, i.e., FRAP, TEAC, and ORAC (Figure 6).

The results obtained by the TEAC and ORAC assays univocally show that the fractions enriched in soluble-conjugated phenolic acids and flavonoids possess a higher AA compared to the free fraction (Figure 6A,B). FRAP provided the same results (data not shown). Although this may derive, at least in part, from the above-cited overestimation due to the formation of 5-hydroxymethyl-2-furfural, which displays AA [34], it has been widely demonstrated that soluble-conjugated polyphenols and/or feruloylated/coumaroylated short oligosaccharides are able to greatly contribute to the antioxidant capacity of grains [56,57]. As concerns the comparison among samples, the results obtained with the TEAC/FRAP and ORAC assays are slightly different. For all fractions (free, AH, BH), the former assay (Figure 6A; FRAP data not shown) revealed that TD had a slightly, but significantly, higher AA compared to TI and to both Regalona extracts, which, conversely, did not differ from each other. According to ORAC, no differences among samples were observed in the free fraction, while for the AH one, TD was confirmed to have the highest AA; no differences were found between the two Regalona samples grown in Chile and in Italy (Figure 6B). In the BH fraction, a statistically significant difference emerged between the Italian-grown Regalona and both RC and TD, even though the biological relevance of this difference is questionable. Thus, it can be concluded that the higher amount of flavonol derivatives in Danish-grown Titicaca might contribute to the higher antioxidant activity, given the strong redox capacity associated to this subgroup of flavonoids.

For an overall comparison among samples, the total phenolic index (TPI) and the total antioxidant index (TAI) were calculated as the sum of the individual phenolic compounds and AA, respectively, detected in all fractions.

**Figure 7:**
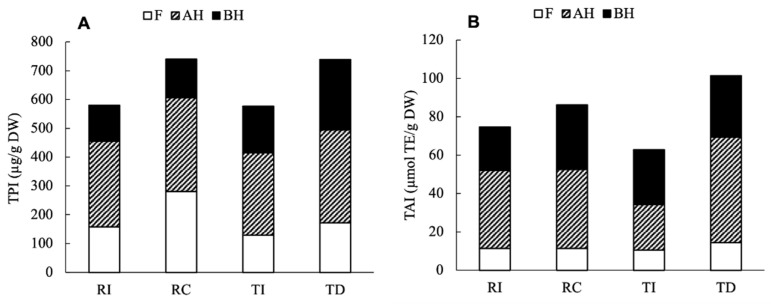
Total phenolic index (TPI, **A**) and total antioxidant index (TAI, **B**) in the four seed extracts of quinoa. TPI was calculated as the sum of individual phenolic compounds detected, and TAI as the sum of ORAC-assayed AA in each fraction.

As shown in Figure 7A, despite a different distribution between the free and soluble conjugated forms, the results clearly indicate that the Chilean Regalona and the Danish Titicaca show a very similar TPI, while seeds of these cultivars grown in Italy respond to the different climatic/environmental conditions with a 20% lower accumulation of the main phenolic compounds compared to their counterparts. The results of the two-way ANOVA indicate that the area of cultivation significantly contributed to the variance of TPI (78%, *p* = 0.005), while neither the genotypes nor their interaction had a significant impact on the variation (*p* = 0.13 and 0.56, respectively). As for the TAI resulting from the ORAC assay (Figure 7B), it was only slightly lower (15%) in RI compared to RC, while a greater difference (40%) was observed between TD and TI; similar results were found for the TEAC assay (data not shown). These results confirm that the total antioxidant activity only weakly correlates with the TPI, as it is more tightly related to the specific composition in antioxidants; in fact, single compounds or classes of compounds can provide a greater contribution compared to others. The two-way ANOVA indicated that the total antioxidant index did not vary significantly depending on genotype (*p* = 0.61), while the cultivation area, as well as its interaction with genotype, provided a significant contribution (*p <* 0.0001 and *p* = 0.0031, respectively).

In conclusion, the present results suggest that, both in terms of phenolic profiles and AA, genotype-dependent differences are not very relevant as far as Titicaca and Regalona are concerned, both of which are adapted to temperate climates, belong to the coastal ecotype, and were bred from gene pools originating from southern/central Chile. The results also indicate that agroecological conditions can, to some extent, alter these phytochemical profiles and the biological activities; in particular, light/UV-B intensity may have contributed to higher flavonol levels in seeds from Denmark [58,59]. Nonetheless, the changes are quite small and corroborate previous data [25], demonstrating that both Titicaca and Regalona can be successfully cultivated in Italy, where they maintain good growth, productivity, and nutritional properties.

## 3. Materials and Methods

### 3.1. Chemicals and Reagents

The following chemicals and reagents were purchased from Sigma-Aldrich Italia (Milan, Italy): Folin–Ciocalteu’s phenol reagent, 6-hydroxyl-2,5,7,8-tetramethyl-chroman-2-carboxylic acid (Trolox), 2,2-azinobis (3-ethylbenzothiazoline-6-sulfonic acid; ABTS), AlCl_3_, NaNO_2_, FeCl_2_, FeCl_3_, FerroZine^®^, fluorescein, 2,2-azobis(2-amidinopropane) dihydrochloride (AAPH), phosphoric acid (85–87%, *w*/*w*), hydrochloric acid (37%, *w*/*w*), monobasic sodium phosphate (>98%), sodium hydroxide beads (>98%), and HPLC-grade solvents. Pure standards of phenolic acids (4-hydroxybenzoic, gallic, caffeic, chlorogenic, ferulic, *p*-coumaric, sinapic, syringic, trans-cinnamic, and vanillic acids) and flavonoids (quercetin, quercetin-3-*O*-glucoside, quercetin-3-*O*-rutinoside, quercetin-3-*O*-galactoside, kaempferol, kaempferol-3-*O*-rutinoside, catechin, epicatechin, daidzein, and genistein) were purchased from Extrasynthese (Genay Cedex, France). The IUPAC names for these compounds are indicated in Table S1. All standards (>99.5% purity in powder form) were prepared as stock solutions at 1 mg/mL in methanol and stored in the dark at −18 °C for less than three months.

### 3.2. Plant Material and Extraction Procedure

Seeds of quinoa cultivars Regalona-Baer (opaque white) and Titicaca (pale yellow) harvested in 2017 were used. Seeds of cv. Regalona collected from plants grown in Italy (RI) were purchased from Dall’Ara & Lolli farm (Campiano, Ravenna, Italy; 44°18′10″ N, 12°12′07″ E; mean annual temperature 14.4 °C, max 19.0 °C, min 8.3 °C; total annual precipitation 646.0 mm), while seeds of the same cultivar grown in Chile (RC) were purchased from the seed company Semillas Baer (Temuco, Chile; 38°44′23″ S, 72°36′00″ W; mean annual temperature 10.9 °C, max 17.3 °C, min 5.9 °C; total annual precipitation 1355.2 mm). Seeds of quinoa cv. Titicaca grown in Italy (TI) were kindly supplied by D. Vanuzzi (Tuttoquinoa) and collected from plants grown in Sale (Alessandria, Italy; 44°58′54″ N, 8°48′37″ E; mean annual temperature 13.5 °C, max 18.6 °C, min 8.5 °C; total annual precipitation 501.5 mm), while Titicaca seeds from plants grown in Denmark (TD) were kindly supplied by S. Jacobsen (Copenhagen, Denmark; 55°38′46″ N, 12°17′53″ E; mean annual temperature 8.9 °C, max 19.2 °C, min −3.4 °C; total annual precipitation 848.8 mm).

The extractions were performed in triplicate, following the procedure described by Tang et al. [32], with slight modifications. Seeds were ground in a knife mill for 4 × 30 s (A11 basic, IKA Werke GmbH & Co. KG, Staufen, Germany) and in a mortar to obtain a fine and homogeneous powder. The seed powder was then subjected to the “coning and quartering” sampling procedure, and three technical replicates were carried out. A 3-g aliquot of fine powdered sample was transferred in a 50-mL tube, mixed with 10 mL of 70% MeOH acidified with HCl (0.1%, *v*/*v*), and kept on an orbital shaker (Duomax 1030, Heidolph Instruments, Schwalbach, Germany) at 200 rpm for 3 h at RT; after a 15-min ultrasound extraction (Elma Schmidbauer GmbH, Singen, Germany), the mixture was centrifuged for 30 min at 4000 rpm, and the supernatant, containing all extractable compounds, was collected. The procedure was repeated twice, and supernatants were combined to form the crude extract (CE). 

#### Preparation of Free (F), Base-Hydrolysed (BH), and Acid-Hydrolysed (AH) Soluble Fractions

To obtain the free (F) fraction, the CE was evaporated to dryness, re-suspended with 2 mL of acidified water (pH = 2), and subsequently extracted with 2 mL of a diethyl ether/ethyl acetate mixture (1:1, *v*/*v*) three times. The organic phases were merged, evaporated to dryness, re-suspended in 2 mL of 70% MeOH, filtered through 0.2-μm nylon syringe filters, and stored at −80 °C until analysis. 

The aqueous phases were also pooled and subjected to base and acid hydrolyses to obtain the base-hydrolysed (BH) and acid-hydrolysed (AH) fractions, respectively. A 2-mL volume of 10 N NaOH was added to 8 mL of aqueous phase, and the mixture was stirred for 1 h under N_2_ flow and brought to pH 2 with concentrated HCl. The resulting solution was then subjected to the extraction with diethyl ether/ethyl acetate mixture as previously described. The combined organic phases were evaporated to dryness, re-suspended with 2 mL of 70% MeOH, filtered through 0.2-μm nylon syringe filters, and stored at −80 °C until analysis.

The same volume of the aqueous phase was subjected to acid hydrolysis, by adding 1.6 mL of 12 N HCl, and the mixture was stirred for 1 h at 85 °C in a water bath. The resulting solution was extracted three times as previously described, and the combined organic phases were evaporated to dryness, re-suspended with 2 mL of 70% MeOH, filtered through 0.2-μm nylon syringe filters, and stored at −80 °C until analysis.

### 3.3. Determination of Total Phenolic Content and Total Flavonoid Content

The total phenolic content (TPC) assay was carried out using the Folin–Ciocalteu reagent according to the procedure described by Singleton and Rossi [60], with modifications. A mixture containing 100 µL of diluted extract fractions or standard and 440 µL of Folin–Ciocalteu’s reagent (diluted 1:10 with water) was incubated for 10 min at RT. Then, 440 µL of 7.5% sodium carbonate was added and the mixture was incubated in the dark for 60 min at RT. Gallic acid was used as a standard, and a calibration curve was built, in the 1–25 ppm range. Absorbance was measured at 765 nm using a double-beam spectrophotometer (V-630 Jasco, Jasco Europe s.r.l., Cremella, Italy) and used to calculate TPC, expressed as mg gallic acid equivalents (GAE) g^−1^ DW of seed powder.

The total flavonoid content (TFC) assay was carried out according to the procedure described by Zou et al. [61], with modifications. A mixture containing 100 µL of diluted extract fractions or standard and 440 µL of 0.066 M NaNO_2_ was left to react for 5 min at RT. Then, 60 µL of 0.75 M AlCl_3_ was added, and the mixture was incubated for 5 min. Lastly, 400 mL of 0.5 M NaOH was added, and the mixture was incubated for 6 min at RT. The absorbance was measured at 510 nm, and the TPC of samples was calculated by interpolating with the calibration curve built with catechin as a standard, in the 1–25 ppm concentration range. Results are expressed as mg catechin equivalents (CE) g^−1^ DW of seed powder.

### 3.4. HPLC Determination of Phenolic Compounds

The chromatographic method was adapted from Tang et al. [32], with some modifications. The extracts were injected into a Jasco (Tokyo, Japan) HPLC-DAD system, which consisted of a PU-4180 pump, an MD-4015 PDA detector, and an AS-4050 autosampler. The stationary phase was an Agilent (Santa Clara, CA, USA) Zorbax Eclipse Plus C18 reverse-phase column (100 × 3 mm I.D., 3.5 μm). The mobile phase was a mixture of solvent A (water/formic acid 95/5, *v*/*v*) and solvent B (methanol/acetonitrile 95/5, *v*/*v*), with a composition gradient ranging from 95% to 5% of solvent A and flowing at 0.7 mL/min. Injection volume was 20 µL for all determinations, and analyte detection was carried out with a diode array detector (DAD) by monitoring at 280, 329, and 360 nm. Quantification was performed with pure standards using calibration curves ranging between 1.25 and 30 μg mL^−1^ (r^2^ ≥ 0.9634).

### 3.5. In Vitro Antioxidant Activity Assays

The ABTS assay was carried out essentially as described by Thaipong et al. [62]. After incubating 950 µL of 7.4 mM 2,2-azinobis(3-ethylbenzothiazoline-6-sulfonic acid) (ABTS) in methanol with either 50 µL of methanolic Trolox solution at different concentrations (0.05–1.00 mM), methanol (blank solution), or the diluted sample in the dark at RT for 2 h, the absorbance of the solution was read at 734 nm in the V630 spectrophotometer. Calibration curves were set up by plotting the discoloration ratio (i.e., [Abs _without TX_/Abs _with TX_] − 1) as a function of Trolox concentration. The antioxidant capacity of the sample, expressed as Trolox equivalents (TEs), was calculated by interpolating with the calibration curve. The oxygen radical absorbance capacity (ORAC) assay was carried out on sample fractions essentially as described by Moore et al. [63], using a Viktor X3 multilabel plate reader (Perkin Elmer, Turku, Finland). Trolox equivalents (TEs) were calculated from the relative area under the curve of the emission intensity vs. time plots.

The ferric reducing antioxidant power-ferrozine (FRAP-FZ) assay was performed as described by Mandrone et al. (2015) [64].

### 3.6. Statistical Analysis

Statistical analysis was performed using GraphPad (San Diego, CA, USA) Prism Software v. 5.0. Comparison among samples was conducted through one-way analysis of variance (ANOVA) with Tukey’s multiple comparison test, and values of *p* ≤ 0.05 were considered statistically significant. Two-way ANOVA was carried out to analyse the genotype x cultivation area interaction.

## Data Availability

Data presented in this study are available upon request.

## Supplementary Materials

The following are available online at https://www.mdpi.com/article/10.3390/plants10061046/s1, Table S1: Common and IUPAC names of the phenolic compounds analysed in the study.
