# Supplementary material for: Free and Conjugated Phenolic Profiles and Antioxidant Activity in Quinoa Seeds and Their Relationship with Genotype and Environment
Source: Plants (Basel). 2021 May 21;10(6):1046. doi: 10.3390/plants10061046 (PMC8224317; doi:10.3390/plants10061046)
Supplement: Supplementary file 1 [file plants-10-01046-s001.zip › plants-1214915-supplementary.pdf]

Table 1S. Common and IUPAC names of the phenolic compounds analysed in the study.

Common name	IUPAC name
4-Hydroxybenzoic acid or *p*-Salycilic acid	4-Hydroxybenzoic acid
Gallic acid	3,4,5-Trihydroxybenzoic acid
Caffeic acid	(*E*)-3-(3,4-Dihydroxyphenyl)prop-2-enoic acid
Chlorogenic acid	(1*S*,3*R*,4*R*,5*R*)-3-[(*E*)-3-(3,4-Dihydroxyphenyl)prop-2-enoyl]oxy-1,4,5-trihydroxycyclohexane-1-carboxylic acid
Ferulic acid	(*E*)-3-(4-Hydroxy-3-methoxyphenyl)prop-2-enoic acid
p-Coumaric acid	(*E*)-3-(4-Hydroxyphenyl)prop-2-enoic acid
Sinapic acid	(*E*)-3-(4-Hydroxy-3,5-dimethoxyphenyl)prop-2-enoic acid
Syringic acid	4-Hydroxy-3,5-dimethoxybenzoic acid
trans-Cinnamic acid	(*E*)-3-Phenylprop-2-enoic acid
Vanillic acid	4-Hydroxy-3-methoxybenzoic acid
Quercetin	2-(3,4-Dihydroxyphenyl)-3,5,7-trihydroxychromen-4-one
Isoquercetin or Quercetin-3-*O*-glucoside	2-(3,4-Dihydroxyphenyl)-5,7-dihydroxy-3-[(2*S*,3*R*,4*S*,5*S*,6*R*)-3,4,5-trihydroxy-6-(hydroxymethyl)oxan-2-yl]oxychromen-4-one
Rutin or Quercetin-3-*O*-rutinoside	2-(3,4-Dihydroxyphenyl)-5,7-dihydroxy-3-[(2*S*,3*R*,4*S*,5*S*,6*R*)-3,4,5-trihydroxy-6-[[(2*R*,3*R*,4*R*,5*R*,6*S*)-3,4,5-trihydroxy-6-methyloxan-2-yl]oxymethyl]oxan-2-yl]oxychromen-4-one
Hyperoside or Quercetin-3-*O*-galactoside	2-(3,4-Dihydroxyphenyl)-5,7-dihydroxy-3-[(2*S*,3*R*,4*S*,5*R*,6*R*)-3,4,5-trihydroxy-6-(hydroxymethyl)oxan-2-yl]oxychromen-4-one
Kaempferol	3,5,7-Trihydroxy-2-(4-hydroxyphenyl)chromen-4-one
Nicotiflorin or Kaempferol-3-*O*-rutinoside	5,7-Dihydroxy-2-(4-hydroxyphenyl)-3-[(2*S*,3*R*,4*S*,5*S*,6*R*)-3,4,5-trihydroxy-6-[[(2*R*,3*R*,4*R*,5*R*,6*S*)-3,4,5-trihydroxy-6-methyloxan-2-yl]oxymethyl]oxan-2-yl]oxychromen-4-one
Catechin	(2*R*,3*S*)-2-(3,4-Dihydroxyphenyl)-3,4-dihydro-2*H*-chromene-3,5,7-triol
Epicatechin	(2*R*,3*R*)-2-(3,4-Dihydroxyphenyl)-3,4-dihydro-2*H*-chromene-3,5,7-triol
Daidzein	7-Hydroxy-3-(4-hydroxyphenyl)chromen-4-one
Genistein	5,7-dihydroxy-3-(4-hydroxyphenyl)chromen-4-one
